# Effects of forest bathing versus indoor gym exercise on exercise-related boredom: a randomized crossover trial with repeated measures

**DOI:** 10.3389/fpsyg.2026.1827928

**Published:** 2026-05-22

**Authors:** Haolan Li, Yu Fang, Bingshan Li, Weimin Zhang, Pengpeng Dong

**Affiliations:** 1 Anhui Lvhai Vocational College of Business, Hefei, China; 2 Department of Public Culture, Nanchang Health Vocational and Technical College, Nanchang, China; 3 Faculty of Education, Nanchang Vocational University, Nanchang, China

**Keywords:** attention restoration, control value theory, crossover design, exercise adherence, forest bathing, green exercise boredom, perceived restorativeness, shinrin yoku

## Abstract

**Objective:**

Exercise boredom is a pervasive barrier to physical activity adherence; however, no study has examined it as a primary outcome using a multidimensional boredom measure with temporal tracking across exercise settings. This study compared forest bathing and indoor gym exercise on exercise-related boredom, affective responses, perceived restorativeness, and adherence intention.

**Design:**

This is a randomized, counterbalanced crossover trial with repeated measures at four time points.

**Methods:**

A total of 34 healthy university students (21 male and 13 female students; mean age = 21.3 years) completed two intensity-matched, 30-min walking sessions (forest-path and indoor treadmill conditions) in a counterbalanced order, separated by a washout period of 7- to 10-days between them. The primary outcome was exercise boredom, assessed using an 8-item adapted Multidimensional State Boredom Scale (MSBS; Cronbach’s *α* = 0.87–0.89). Data were analyzed using a 2 (condition) × 4 (time) repeated-measures analysis of variance (ANOVA) with Greenhouse–Geisser correction.

**Results:**

A significant condition × time interaction emerged for boredom [*F*(1.75, 57.62) = 7.17, *p* = 0.003, partial *η*^2^ = 0.179]. Post-exercise boredom was lower after forest bathing compared to gym exercise [mean difference = 0.60, 95% confidence interval (0.17, 1.03), *d* = 0.49]. Boredom decreased during forest walking but increased during treadmill walking (*p* < 0.001). Forest bathing resulted in higher affective valence (*d* = 0.43), greater perceived restorativeness (*d* = 1.84), and stronger adherence intention (*d* = 0.37). Ratings of perceived exertion confirmed equivalent exercise intensity.

**Conclusion:**

Exercise boredom is not merely an individual disposition but a context-sensitive response that is environmentally modifiable. Nature-based exercise warrants further investigation as a strategy for sustaining physical activity engagement.

## 1 Introduction

A university student steps onto a gym treadmill. Within minutes, tedium sets in, and the remaining time feels interminable. By the following week, the gym membership is left unused. This scenario illustrates exercise-related boredom, a pervasive yet neglected barrier to physical activity adherence. Exercise is often perceived not as challenging but as monotonous, and the resulting boredom may drive dropout, regardless of their motivation or fitness levels. Despite decades of research on psychological determinants of adherence, boredom has remained conspicuously absent from the empirical agenda in sport and exercise psychology (Bieleke et al., 2025; Wolff et al., 2021).

Physical inactivity remains a global health concern (World Health Organization, 2022). Although the health benefits of regular exercise are well established (Warburton and Bredin, 2017), adherence remains persistently low: 40–65% of individuals who begin structured programs discontinue within 6 months (Robison and Rogers, 1994; Sperandei et al., 2016). Boredom and lack of enjoyment are commonly reported barriers (Ferreira Silva et al., 2022; Salmon et al., 2003). Critically, adherence is not solely a motivational problem; it is also an experiential one. If the exercise itself feels boring, even well-motivated individuals may disengage. However, boredom has received remarkably little direct investigation as a modifiable psychological barrier.

Boredom is an aversive state characterized by insufficient stimulation, perceived meaninglessness, and a desire for more satisfying engagement (Eastwood et al., 2012; Elpidorou, 2018). Control-value theory (CVT) (Pekrun, 2006) provides a rigorous framework: boredom arises when an individual perceives low control over and low subjective value in an ongoing activity. In a recent study by Bieleke et al. (2025), the first application of CVT to exercise was presented, involving 667 recreational endurance athletes. The study found that lower perceived challenge and subjective value were robust antecedents of exercise boredom, which in turn led to reduced training frequency. These findings establish CVT as the most promising theoretical approach for understanding why certain exercise contexts may be inherently more boring.

Attention restoration theory (ART) (Kaplan and Kaplan, 1989; Kaplan, 1995) offers a complementary environmental mechanism, identifying four restorative properties of natural environments: soft fascination, being away, extent, and compatibility (Ohly et al., 2016; Stevenson et al., 2018). Ulrich’s (1983) stress reduction theory (SRT) adds that natural scenes elicit innate positive affect. Integrating these perspectives with CVT yields a mechanistic model: attention restoration theory and stress reduction theory do not compete with CVT but rather operate through its appraisal processes. Specifically, soft fascination, as described in attention restoration theory, may enhance the perceived value of the exercise experience, while the environmental variability and exploratory affordances of natural settings may increase perceived control over pace, route, and sensory engagement. Together, these altered appraisals should reduce boredom according to CVT predictions. CVT thus provides the primary explanatory framework for the present study, with attention restoration theory and stress reduction theory specifying the upstream environmental features through which CVT appraisal dimensions may be altered.

Meta-analytic reviews of green exercise indicate modest but reliable improvements in affective valence compared to indoor exercise; however, the heterogeneity is high among studies, and the effects on other outcomes are inconsistent (Lahart et al., 2019; Li et al., 2022; Thompson Coon et al., 2011). Specifically, in the context of forest bathing, meta-analyses document significant reductions in cortisol and anxiety levels with large effect sizes (*g* > 0.80), although these studies have focused almost exclusively on stress and mood outcomes; boredom has not been isolated as a distinct experiential construct in this literature (Antonelli et al., 2019; Kotera et al., 2022; Yeon et al., 2021).

Three gaps, spanning conceptual, methodological, and measurement domains, persist in the literature. First, in terms of measurement, no study has examined exercise boredom as a primary dependent variable with a multidimensional validated boredom measure and temporal tracking. Second, at the methodological level, within-subject crossover designs remain rare in green exercise research (Laezza et al., 2024). Third, at the conceptual level, the green exercise literature has not been integrated with CVT’s framework for exercise boredom, leaving the mechanisms linking environment to boredom unexplored.

Laezza et al. (2025) compared walking in natural, urban, and indoor environments using a crossover design with 25 young men and found that boredom increased in indoor settings but decreased in nature (Laezza et al., 2025). Rather than serving as a straightforward replication, the present study constitutes the first theoretically integrated test of exercise boredom within a combined CVT and environmental psychology framework. It extends the work of Laezza et al. (2025) in several critical ways: we employed a multi-item boredom scale (rather than a single item in a mood profile), included women and men, measured perceived restorativeness and nature-relatedness, and tested the condition × time interaction via repeated-measures analysis of variance (ANOVA) with sphericity correction. These extensions address the measurement precision and analytic rigor limitations of prior work.

Drawing on CVT as the primary framework, we tested the following hypotheses:

*H1:* A significant condition × time interaction for boredom, with lower boredom in the forest post-exercise.

*H2:* More positive affective valence in the forest condition.

*H3:* Stronger adherence intentions following the forest condition.

*H4:* Substantially higher perceived restorativeness in the forest.

## 2 Methods

### 2.1 Trial design and registration

**Figure 1:**
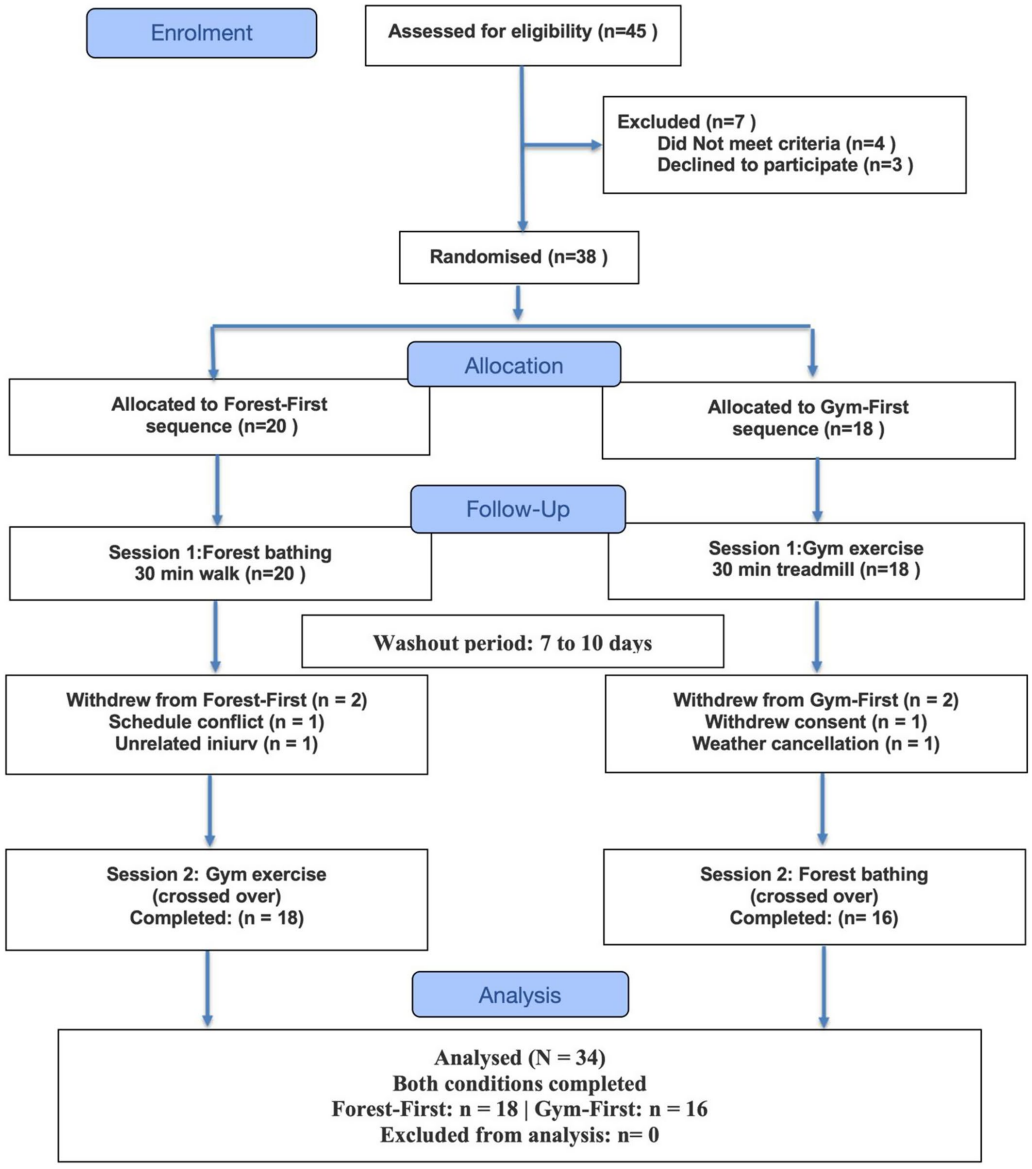
CONSORT flow diagram.

This study employed a randomized, counterbalanced crossover design with two within-subject conditions and repeated measures at four time points. A 7- to 10-day washout period separated conditions. The study was approved by the institutional review board of Nanchang Vocational University (Protocol No. NCVU 2025RT 2,511) and conducted in accordance with the Declaration of Helsinki. Reporting follows CONSORT guidelines for crossover trials (Dwan et al., 2019); (see Figure 1).

### 2.2 Participants

*A priori* power analysis (G*Power 3.1, Heinrich-Heine-Universität Düsseldorf, Düsseldorf, Germany) (Faul et al., 2009) targeting dz = 0.50 at *α* = 0.05 with 80% power yielded a minimum of 34 completers. This provides adequate power for the primary comparison, but the sample may lack the power needed to detect small interaction effects or moderation; this is acknowledged in the Discussion section.

Of 45 participants screened, 38 enrolled in the study, and 34 completed both conditions (89.5% retention; Figure 1). Among the participants, there were 21 men and 13 women [mean (M) age = 21.3, standard deviation (SD) = 1.6; mean body mass index = 23.2 kg/m^2^, SD = 3.6; exercise frequency: 3.1 sessions/week]. All participants were urban-dwelling university students residing in Nanchang, a city of approximately 6.4 million people in southeastern China (Table 1).

**Table 1:** Participant demographics (*N* = 34).

Variable	M (SD) or *n* (%)	Range
Age (years)	21.3 (1.6)	18–25
Gender: male	21 (61.8%)	
Gender: female	13 (38.2%)	
BMI (kg/m^2^)	23.2 (3.6)	16.3–31.8
Exercise (d/week)	3.1 (1.2)	1–5
NR-6	2.94 (0.65)	1.40–4.76
Forest first	18 (52.9%)	
Gym first	16 (47.1%)	

M, mean; SD, standard deviation; *n*, number; BMI, body mass index; NR-6, nature relatedness scale short form.

### 2.3 Interventions

#### 2.3.1 Forest bathing

Participants engaged in a 30-min walk along a 2.2-km loop trail in Meiling National Forest Park (which features a subtropical evergreen forest with a canopy predominantly comprising *Cinnamomum camphora* and *Pinus massoniana*). The park is located approximately 15 km from the campus in Nanchang, Jiangxi, China. During the walk, participants maintained a pace corresponding to 40–60% of their heart rate reserve (HRR) via Polar H10 (Polar Electro Oy, Kempele, Finland). Consistent with shinrin yoku practice (Li, 2022; Park et al., 2010), participants were encouraged to focus on natural stimuli without structured mindfulness activities.

#### 2.3.2 Gym exercise

Participants also engaged in a30-min treadmill walk (Technogym Excite Run 1,000, Technogym S.p.A., Cesena, Italy) at the same HRR target in a windowless gymnasium. No music, television, or entertainment was provided. This intentional “bare” design, consistent with previous controlled comparisons, isolates the environmental variable. However, it differs substantially from typical gym conditions, where music, video screens, or social interactions are usually available. As a result, this design choice may overestimate the difference in boredom compared to naturalistic gym settings. The implications for ecological validity are addressed further in the Discussion section.

Environmental conditions included a mean temperature of 21.8 °C (SD = 3.3) and humidity of 63.3% (SD = 12.4) in the forest. In contrast, the gym had a mean temperature of 24.1 °C (SD = 1.2). The temperature in the forest was not correlated with post-exercise boredom (*r* = 0.04, *p* = 0.838).

### 2.4 Measures

All instruments used validated Chinese versions.

#### 2.4.1 Exercise boredom (primary)

An 8-item adapted Multidimensional State Boredom Scale (MSBS) was used (Fahlman et al., 2013); Responses are given on the 7-point scale (Cronbach’s *α* = 0.87–0.89). Exploratory principal axis factoring yielded one dominant factor (52% variance), supporting unidimensionality. However, independent validation is still needed.

#### 2.4.2 Affective valence

The Feeling Scale (Hardy and Rejeski, 1989), an 11-point bipolar scale, was used at four time points.

#### 2.4.3 Felt Arousal

The Felt Arousal Scale (Svebak and Murgatroyd, 1985) is a 6-item tool that assesses arousal levels at pre-, mid-, and post-exercise.

#### 2.4.4 Enjoyment

A new short, theory-driven, version of the physical activity enjoyment scale (PACES) (8 items) (Kendzierski and DeCarlo, 1991) was administered post-exercise only (Cronbach’s *α* = 0.91).

#### 2.4.5 Perceived Restorativeness

The Perceived Restorativeness Scale (PRS) (Hartig et al., 1997), 7-point scale, (Cronbach’s *α* = 0.85) was also administered post exercise.

#### 2.4.6 Adherence intention

Adherence intention was assessed using three items derived from the Theory of Planned Behavior questionnaire guidelines (Ajzen, 2006), rated on a 7-point Likert scale (1 = strongly disagree, 7 = strongly agree; Cronbach’s *α* = 0.83). A sample item is “I intend to continue this type of exercise in the coming weeks.”

#### 2.4.7 Nature relatedness

The short form of the Nature Relatedness (NR-6) scale (Nisbet and Zelenski, 2013) was administered at baseline only (M = 2.94, SD = 0.65; Cronbach’s *α* = 0.78).

#### 2.4.8 Physiological measures

Physiological measures included heart rate (HR; Polar H10) and the rating of perceived exertion (RPE; Borg 6–20), collected at mid- and post-exercise.

#### 2.4.9 Awareness probe

After the second session, participants reported what they believed the study investigated. Only 3 (8.8%) out of 34 participants identified boredom as the primary outcome.

### 2.5 Procedure

Data were collected from 15 September to 2 November 2025. Baseline assessment included demographics, NR-6, and randomization (blocks of 4). Each session included a 5-min rest, pre-exercise measures, a 30-min walk (mid-exercise measures at 15 min, ~90-s pause), an immediate post-exercise battery, and a 30-min follow-up.

### 2.6 Data analysis

Primary analysis: 2 (condition) × 4 (time) repeated-measures ANOVA. Mauchly’s test assessed sphericity, where violated, Greenhouse–Geisser (GG) corrected degrees of freedom and *p* values are reported. Holm-corrected pairwise comparisons followed the interaction. Effect sizes: partial eta squared (ANOVA), Cohen’s *d* (paired), with 95% CIs. Secondary outcomes: paired *t*-tests. Sensitivity analyses examined order effects, gender (including exploratory three-way condition × time × gender interactions), temperature, NR-6 moderation (Cronbach’s *α* = 0.05).

## 3 Results

### 3.1 Participant flow and preliminary analyses

See Figure 1 (CONSORT). Of the 45 participants screened, 34 completed both conditions. No significant order effects were observed (forest: *p* = 0.494; gym: *p* = 0.876).

### 3.2 Manipulation check

The RPE did not differ mid-exercise [forest 11.6 vs. gym 11.7; *t*(33) = 0.08, *p* = 0.934, *d* = 0.01, 95% CI (0.72, 0.66)] or post-exercise [12.5 vs. 12.7; *t*(33) = 0.50, *p* = 0.620, *d* = 0.09, 95% CI (1.16, 0.70)]. Mean exercise HR was marginally higher in the gym (112.5 vs. 109.9 bpm; *p* = 0.077), biasing against our hypotheses. This small physiological difference likely reflects slight variation in terrain and self-pacing rather than a meaningful intensity mismatch.

### 3.3 Primary outcome: exercise boredom (H1)

**Figure 2:**
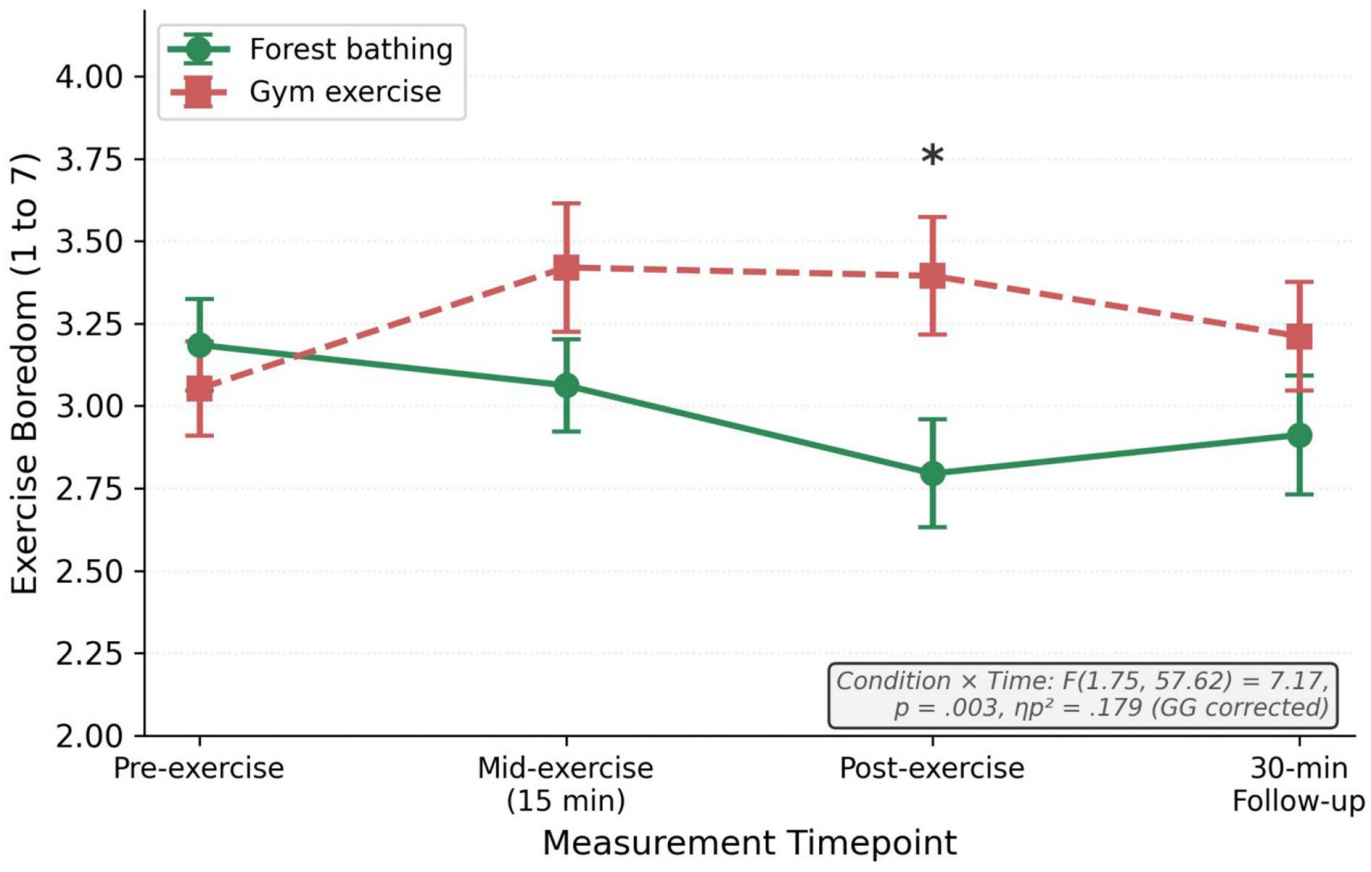
Boredom trajectories by condition. *p_Holm_ < 0.05. Condition × time: *F*(1.75, 57.62) = 7.17, *p* = 0.003, *ηp*^2^ = 0.179 [Greenhouse–Geisser (GG) corrected].

**Figure 3:**
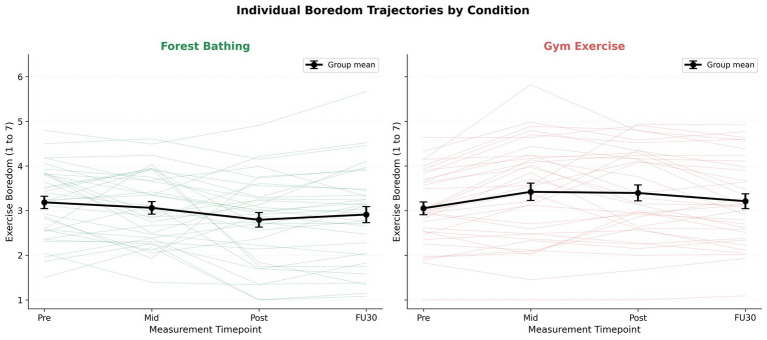
Individual boredom trajectories (thin lines) and group means ± standard error (thick lines). The majority showed the predicted pattern (decrease in forest, increase in gym), with greater variability in the gym condition.

Mauchly’s test indicated sphericity violations for both time (*W* = 0.36, *p* < 0.001) and condition × time (*W* = 0.30, *p* < 0.001); The GG corrected results are reported. The repeated-measures ANOVA revealed a non-significant condition main effect, *F*(1, 33) = 3.25, *p* = 0.081, partial eta squared = 0.090, and a non-significant time main effect, *F*(1.83, 60.49) = 2.54, *p* = 0.092, partial eta squared = 0.071 (GG epsilon = 0.61). The condition × time interaction was significant, *F*(1.75, 57.62) = 7.17, *p* = 0.003, partial eta squared = 0.179 (GG epsilon = 0.58), confirming divergent boredom trajectories (Figures 2, 3).

Holm-corrected pairwise comparisons (Table 2): Pre-exercise, *pHolm* = 0.325 (no baseline difference). Mid-exercise, *t*(33) = 1.97, *pHolm* = 0.171, *d* = 0.34, 95% CI [0.01, 0.73]. Post-exercise, *t*(33) = 2.85, *pHolm* = 0.030, *d* = 0.49, 95% CI [0.17, 1.03] (forest M = 2.79 vs. gym M = 3.39). Follow up, *pHolm* = 0.302, *d* = 0.25. Change scores confirmed the divergence: the forest condition decreased by 0.39 (SD = 0.49), whereas the gym condition increased by 0.34 (SD = 0.54); *t*(33) = 5.57, *p* < 0.001, 95% CI [1.00, 0.47]. Figure 3 displays individual trajectories, and the majority showed the predicted pattern.

**Table 2:** Exercise boredom: RM-ANOVA and Holm-corrected pairwise comparisons.

Source	Pre M (SD)	Mid M (SD)	Post M (SD)	FU30 M (SD)	*F*	*p*	*ηp*^2^	εGG
Forest	3.19 (0.81)	3.06 (0.82)	2.79 (0.96)	2.91 (1.05)				
Gym	3.05 (0.83)	3.42 (1.14)	3.39 (1.04)	3.21 (0.96)				
Condition					3.25	0.081	0.090	n/a
Time					2.54	0.092	0.071	0.61
*C × T*					7.17	0.003	0.179	0.58

GG = Greenhouse–Geisser corrected (Mauchly *p* < 0.001). εGG = GG epsilon; n/a for Condition (2 levels, sphericity not applicable). Time εGG = 0.61; *C* × *T* εGG = 0.58. Holm pairwise: Pre 0.325; Mid 0.171; Post 0.030* [*d* = 0.49, 95% CI (0.17, 1.03)]; FU30 0.302.

εGG, Greenhouse–Geisser epsilon; FU30, 30-min follow-up; CI, confidence interval.

### 3.4 Secondary outcomes (H2)

**Figure 4:**
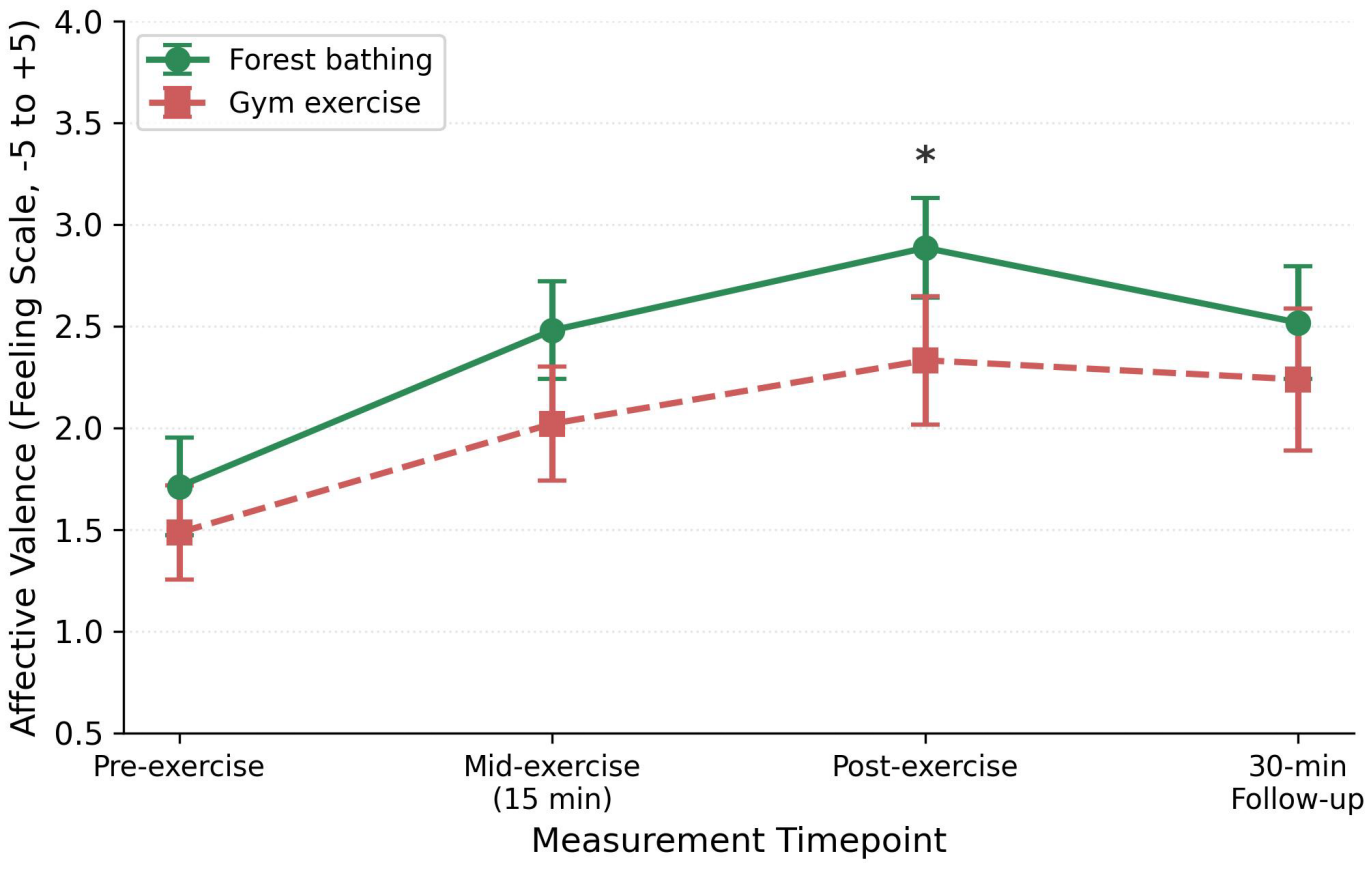
Affective valence (Feeling Scale) trajectories. *p < 0.05.

Post-exercise affective valence was higher after forest bathing (M = 2.89) than after gym exercise [M = 2.33; *t*(33) = 2.51, *p* = 0.017, *d* = 0.43, 95% CI (0.10, 1.00); Figure 4]. Pre- and follow-up differences were not significant. Felt arousal was higher in the gym at mid-exercise (*p* = 0.043, *d* = 0.36). H2 was partially supported.

### 3.5 Post-exercise measures (H3 and H4)

**Figure 5:**
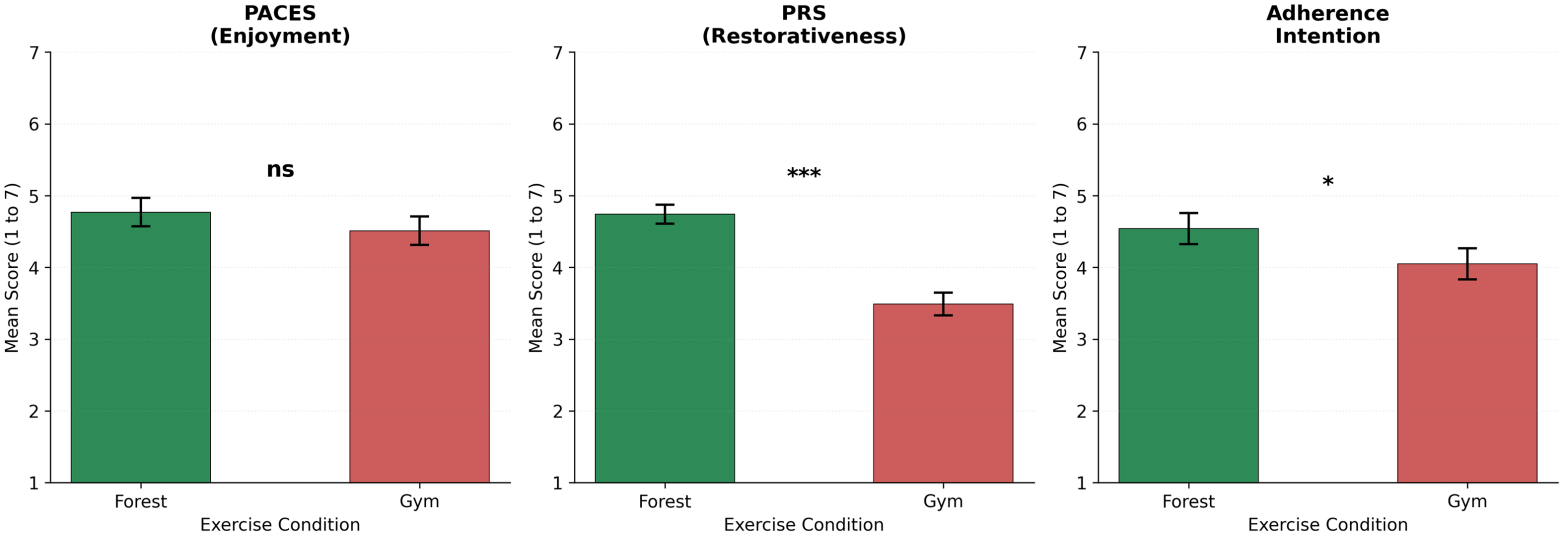
Post-exercise measures. ***p < 0.001; *p < 0.05; ns, not significant.

Post-exercise comparisons are presented in Table 3 and Figure 3. Exercise enjoyment, measured by the Physical Activity Enjoyment Scale (PACES), did not differ significantly between conditions (*p* = .099, *d* = 0.29, 95% CI [−0.05, 0.57]). Perceived restorativeness, measured by the Perceived Restorativeness Scale (PRS), was substantially higher in the forest condition than in the gym condition (forest M = 4.74 vs. gym M = 3.49, *d* = 1.84, *p* < .001, 95% CI [1.02, 1.49]), supporting H4. Adherence intention was also significantly higher following the forest condition (forest M = 4.54 vs. gym M = 4.05, *d* = 0.37, *p* = .039, 95% CI [0.03, 0.96]), supporting H3. (Table 3, Figure 5).

**Table 3:** Post-exercise measures (*N* = 34).

Measure	Forest	Gym	*t*(33)	*p*	*d*	95% CI	*α*
PACES	4.77 (1.16)	4.51 (1.15)	1.70	0.099	0.29	[−0.05, 0.57]	0.91
PRS	4.74 (0.77)	3.49 (0.92)	10.74	<0.001	1.84	[1.02, 1.49]	0.85
Adherence	4.54 (1.27)	4.05 (1.26)	2.15	0.039	0.37	[0.03, 0.96]	0.83
RPE Post	12.5 (2.5)	12.7 (2.2)	0.50	0.620	0.09	[−1.16, 0.70]	—

CI, confidence interval; PACES, physical activity enjoyment scale; PRS, perceived restorativeness scale; RPE, rating of perceived exertion; *α*, Cronbach’s alpha.

### 3.6 Sensitivity analyses

#### 3.6.1 Gender

The boredom effect was consistent across genders (men *d* = 0.36; women *d* = 0.99). An exploratory condition × time × gender test confirmed that the interaction did not differ by gender (*t*(32) = 0.13, *p* = 0.900). The larger effect observed in women should be interpreted cautiously due to the unbalanced subgroups.

#### 3.6.2 Nature relatedness

The NR-6 scale did not moderate the boredom difference (*r* = 0.042, *p* = 0.812; *post hoc* power ~6%).

#### 3.6.3 Environmental covariates

Forest temperature: *r* = 0.04, *p* = 0.838.

#### 3.6.4 Perceived restorativeness and boredom

Within-condition correlations were non-significant (forest *r* = 0.17; gym *r* = 0.12), possibly reflecting a threshold effect, indirect pathway, or range restriction.

## 4 Discussion

The present study provides the first randomized crossover evidence that exercising in a forest environment significantly alters the temporal trajectory of exercise boredom compared to intensity-matched indoor gym exercise. The significant interaction between condition and time point confirmed that boredom progressively decreased during forest walking but increased during treadmill walking, with the largest divergence observed immediately after exercise (*d* = 0.49). This divergent pattern was accompanied by higher affective valence, substantially greater perceived restorativeness, and stronger adherence intentions in the forest condition, while equivalent perceived exertion confirmed that intensity differences did not account for the results. Taken together, these findings advance a broader conceptual point: exercise boredom is not merely a stable individual disposition but a dynamic, context-dependent emotional response that is environmentally modifiable. The exercise environment, far from being a neutral backdrop, functions as an active ingredient in the psychological experience of physical activity. This reconceptualization carries implications for both adherence theory and exercise prescription, as it suggests that modifying where people exercise may be as important as modifying how much or how intensely they exercise.

### 4.1 Exercise boredom through control-value theory

The boredom effect (*d* = 0.49) aligns with the results of Laezza et al. (2025). The present study extends their findings through a multi-item measure, inclusion of women and men, measurement of restorativeness and nature relatedness, and a statistically robust interaction via repeated-measures ANOVA with GG correction. CVT (Bieleke et al., 2025; Pekrun, 2006) provides the primary interpretation. The proposed mechanistic pathway is as follows: the forest environment, through its sensory richness and varied terrain, likely engaged soft fascination (an attention restoration theory construct), which in turn enhanced the perceived value of the exercise experience. Simultaneously, the self-directed pacing and route variability inherent in trail walking may have increased participants’ sense of control. Both enhanced value and enhanced control would reduce boredom according to CVT. By contrast, indoor treadmill walking at a fixed speed in a sensory-impoverished environment is prototypically low on both appraisal dimensions, creating conditions highly conducive to boredom. Importantly, this mechanistic account remains a theoretically informed inference, as we did not directly measure perceived control or value appraisals. Future studies should incorporate momentary assessments of these appraisals during exercise to test the proposed pathway empirically.

### 4.2 The perceived restorativeness effect

The perceived restorativeness effect (*d* = 1.84) was the largest in this study and establishes that the forest was perceived as qualitatively different in precisely the manner predicted by attention restoration theory. The magnitude of this effect substantially exceeds typical green exercise effect sizes reported in meta-analyses, suggesting that the forest trail provided an exceptionally restorative setting. However, within-condition correlations between perceived restorativeness and boredom were not significant, which complicates a simple linear mediation account. Three explanations merit consideration. First, there is a threshold model: once environmental restorativeness exceeds a minimum level, further increments may not linearly reduce boredom. Second, there is an indirect pathway: soft fascination may reduce the self-regulatory effort that produces boredom, operating through attentional engagement rather than through subjective restorativeness ratings. Third, the range restriction in the perceived restorativeness scores for forest bathing may have attenuated the correlations. Distinguishing among these explanations will require process-oriented designs with momentary ecological assessments of both restorativeness and boredom during exercise.

### 4.3 Affective valence, adherence, and the boredom mechanism

The affective valence effect (*d* = 0.43) converges with meta analytic estimates (*d* = 0.46) (Lahart et al., 2019). Exercise enjoyment (PACES) did not reach significance; we did not interpret this trend. Adherence intention was significant (*d* = 0.37). More broadly, these findings suggest that boredom may constitute a missing link in existing adherence models, which have emphasized motivation, self-efficacy, and habit formation but have largely overlooked the experiential quality of exercise itself. If natural environments reduce boredom, and reduced boredom in turn sustains behavioral engagement, then environmental modulation of boredom represents a novel and previously unrecognized pathway in the causal chain from exercise prescription to long-term adherence. Identifying boredom as an environmentally modifiable mediator between exercise context and continuation behavior could meaningfully reshape how exercise interventions are designed. Testing this mediational chain through prospective, multi-session designs should be a priority for future research.

### 4.4 Demand characteristics

Only 8.8% of participants identified boredom as the primary outcome. Enjoyment (a face valid nature benefit) did not reach significance. Pre-exercise boredom was equivalent. These features argue against a purely demand-driven account, although implicit effects cannot be excluded.

### 4.5 Cultural context

This study was conducted involving urban-dwelling Chinese university students in Nanchang, a mid-sized Chinese city. The extent to which these young, educated, urban participants represent broader populations remains uncertain; individuals with greater prior nature exposure or those from rural backgrounds might respond differently. Although shinrin yoku originated in Japan (Li, 2022), forest recreation has deep East Asian roots. Generalizability to Western populations requires cross-cultural replication.

### 4.6 Limitations

Several limitations should be considered. Regarding ecological validity, the most important limitation is the “bare” gym condition, which removed music, videos, and social interactions that characterize the majority of real-world gym environments. This design choice was deliberate, enabling the isolation of the environmental variable, and is consistent with prior controlled comparisons. However, it likely inflated the boredom differential relative to what would be observed in a typical gym. Future studies should compare nature-based exercise against ecologically representative indoor conditions that include typical distractions. Relatedly, the 2.3-degree temperature difference between settings, although not correlated with boredom, cannot be fully dismissed as a confounding factor. Regarding measurement, the adapted MSBS requires independent psychometric validation, the 90-s mid-exercise measurement pause may have disrupted experiential flow, and CVT appraisals (perceived control and value) were not directly measured, limiting mechanistic confirmation. Regarding generalizability, the young urban university student sample may not represent older adults, clinical populations, or individuals from non-urban backgrounds. The single-session design cannot address whether boredom differences persist across repeated exposures. Finally, the sample may be underpowered for detecting small interaction effects or moderation, and we measured adherence intention rather than actual long-term exercise behavior.

### 4.7 Future directions

Future research should pursue four organized priorities. At the theoretical level, direct measurement of CVT appraisals (perceived control and value) during exercise across environments is needed to test the mechanistic pathway proposed above. At the methodological level, virtual reality nature exposure during indoor exercise would permit partial participant blinding, and longitudinal multi-session designs are essential to determine whether boredom differences persist or attenuate with repeated exposure. At the neurocognitive level, functional near infrared spectroscopy could measure engagement in the default mode network during exercise, serving as a neural marker for the soft fascination process proposed by attention restoration theory. On a practical level, extending this work to clinical populations with elevated boredom susceptibility, such as individuals with attention deficit hyperactivity disorder or depression, could help identify groups that may particularly benefit from nature-based exercise prescription.

## 5 Conclusion

This study demonstrates that a forest environment significantly alters the trajectory of exercise boredom compared to intensity-matched indoor exercise. The significant interaction between exercise condition and time, which remains robust even after sphericity correction and multiple comparison adjustments, positions exercise boredom as a context-sensitive, environmentally modifiable emotional response. Nature-based exercise prescription represents a potential strategy that warrants further validation through longitudinal designs with diverse samples and direct measurements of appraisal mechanisms before being recommended as a standard component of exercise prescription.

## Data Availability

The raw data supporting the conclusions of this article will be made available by the authors, without undue reservation.
